# Supplementary material for: Serial amplification of tau filaments using Alzheimer's brain homogenates and C322A or C322S recombinant tau
Source: FEBS Lett. 2025 Sep 30;599(19):2768–78. doi: 10.1002/1873-3468.70141 (PMC12519061; doi:10.1002/1873-3468.70141)
Supplement: Supplementary file 1 — Fig. S1. Purification of C322A 0N3R tau. Fig. S2. In vitro seeded assembly of wild‐type 0N3R tau. Fig. S3. Cryo‐EM analysis of AD‐seeded C322A 0N3R tau. Fig. S4. Cryo‐EM analysis of AD‐seeded C322S 0N3R tau. Table S1. Cryo‐EM data statistics and model validation of second‐generation C322A 0N3R filaments. Table S2. Cryo‐EM data statistics and model validation of second‐generation C322S 0N3R filaments. [file FEB2-599-2768-s001.docx]

**SUPPLEMENTARY INFORMATION**

**Serial amplification of tau filaments using Alzheimer’s brain homogenates and C322A or C322S recombinant tau**

Alessia Santambrogio^1,^*, Sofia Lövestam^2^, Michael A. Metrick II^1,3,^*, Thomas Löhr^1^, Peifeng Xu^1^, Nicholas C. T. Galllagher^1^, Bernardino Ghetti^4^, Byron Caughey^3^, Sjors H. W. Scheres^2^, Michele Vendruscolo^1+^

*^1^Centre for Misfolding Diseases, Yusuf Hamied Department of Chemistry,*

*University of Cambridge, Cambridge, UK*

*^2^MRC Laboratory of Molecular Biology, Cambridge, UK*

*^3^Laboratory of Persistent Viral Diseases, Rocky Mountain Laboratories, Division of Intramural Research, National Institute for Allergy and Infectious Diseases, Hamilton, Montana, USA*

*^4^Departmentof Pathology and Laboratory Medicine, Indiana University School of Medicine, Indianapolis, Indiana, USA*

*Equal contributions

+Correspondence: mv245@cam.ac.uk

Keywords

Alzheimer’s disease; tau; cryo-EM; protein aggregation; RT-QuIC; disease-relevant filament polymorphism

**
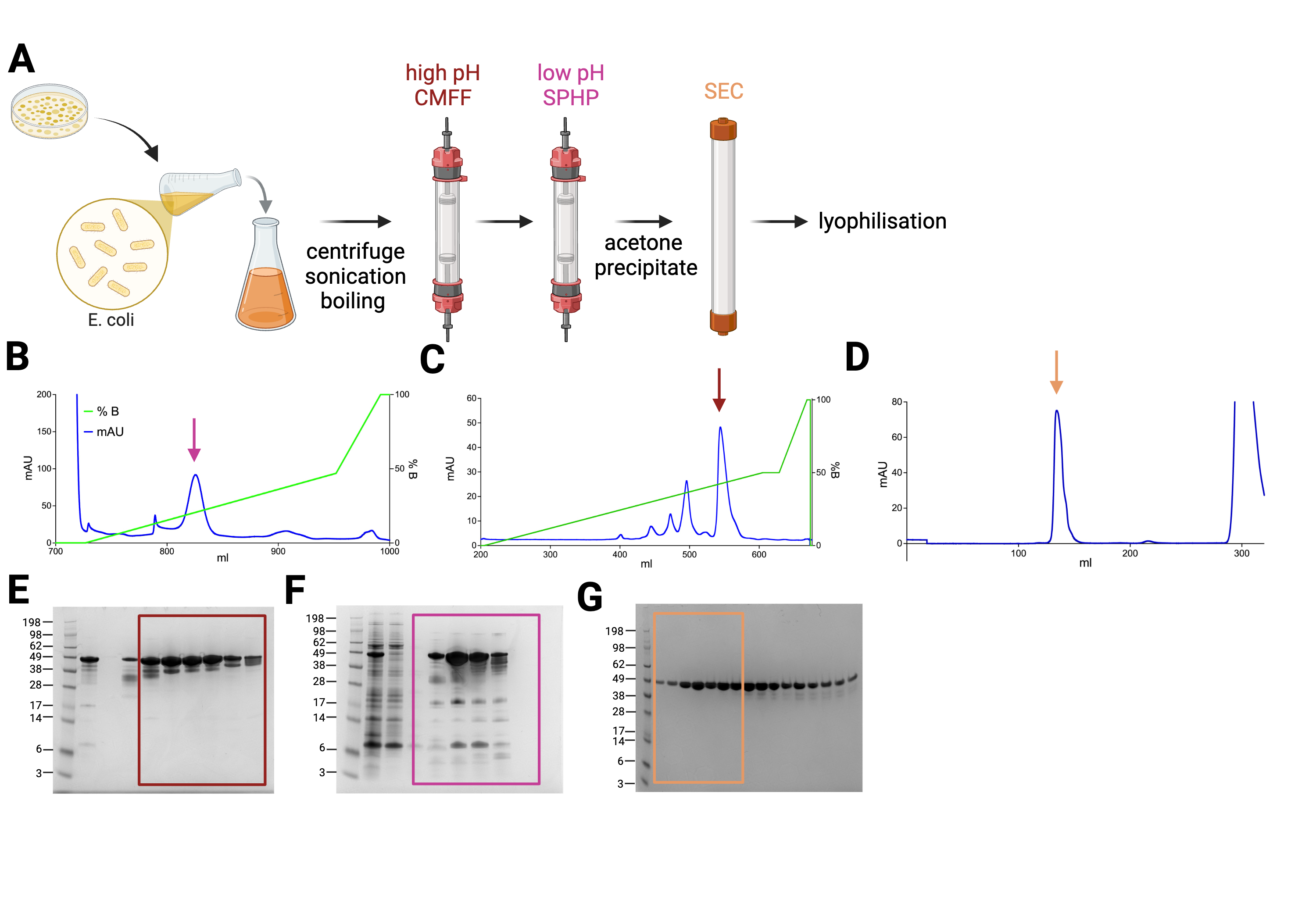
**

**Figure S1. Purification of C322A 0N3R tau**. (**A**) Schematic illustration of the protein purification process, starting from bacterial culture, followed by scale-up, centrifugation, sonication, boiling, CMFF (carboxymethyl fast flow) weak cation exchange chromatography at high pH, SPHP (sulfopropyl high performance) strong cation exchange chromatography at low pH, size exclusion chromatography (SEC), and lyophilization. (**B-D**) Chromatogram spectra of C322A 0N3R tau via cation exchange chromatography (CMFF) at pH 8 over a linear gradient of 0–500 mM NaCl across 30 column volumes (B); cation exchange chromatography (SPHP) at pH 6 over a linear gradient of 0–500 mM NaCl across 30 column volumes (C); size exclusion chromatography (SEC) (D). (**E-G**) Coomassie-stained SDS-PAGE analysis of C322A 0N3R tau after cation exchange chromatography (CMFF) at pH 8 (E); SDS-PAGE after cation exchange chromatography (SPHP) at pH 6 (E); SDS-PAGE after SEC (G).

**Figure S2. *In vitro* seeded assembly of wild-type 0N3R tau**. (**A, B**) ThT fluorescence profiles of AD-seeded and CVD-seeded reactions for round 1 (first-generation) with WT 0N3R tau using either a seed concentration of 0.0001% (**A**) or a higher protein concentration of 50 µM tau (92 µg) (**B**). N=10; AD, Alzheimer’s disease; CVD, cerebrovascular disease.

**Figure S3**. **Cryo-EM analysis of AD-seeded C322A 0N3R tau**. (**A**) Cryo-EM micrograph of AD-seeded C322A 0N3R tau. (**B**) Pie chart showing the distribution of filament types. (**C**) Fourier shell correlation (FSC) curves for: two independently refined half maps (black), the final refined atomic model against the cryo-EM map (red), the atomic model refined in the first half-map (blue), and the refined atomic model in the first half-map against the second half-map (yellow). (**D**) Two-dimensional class averages of AD-seeded C322A 0N3R tau filaments.

**Figure S4**. **Cryo-EM analysis of AD-seeded C322S 0N3R tau**. (**A**) Cryo-EM micrograph of AD-seeded C322S 0N3R tau. (**B**) Pie chart showing the distribution of filament types. (**C**) Fourier shell correlation (FSC) curves for: two independently refined half maps (black), the final refined atomic model against the cryo-EM map (red), the atomic model refined in the first half-map (blue), and the refined atomic model in the first half-map against the second half-map (yellow). (**D**) Two-dimensional class averages of AD-seeded C322S 0N3R tau filaments.

**Table S1: Cryo-EM data statistics and model validation of second-generation**

**C322A 0N3R filaments.**

| **LMB Krios II** | **2nd generation**  **C322A 0N3R**  (EMDB 53527)  (PDB 9R2F) |
| --- | --- |
| **Data acquisition** |  |
| Electron gun | FEG |
| Detector | Falcon 4i |
| Energy filter slit (eV) | na |
| Magnification | 165,000 |
| Voltage (kV) | 300 |
| Electron dose (e-/Å^2^) | 40 |
| Defocus range (μM) | 0.5 to 2.5 |
| Pixel size (Å) | 0.824 |
| **Data processing** |  |
| Initial particle images (no.) | 516293 (manual) |
| Final particle images (no.) | 61103 |
| Helical twist (º) | 179.599 |
| Helical rise (Å) | 2.417 |
| Symmetry imposed | C1 |
| Map resolution FSC 0.143 (Å) | 2.89 |
| **Refinement** |  |
| Initial model used (PDB code) | 6hre |
| Model resolution FSC 0.5 (Å) | 2.8 |
| Map sharpening *B* factor (Å^2^) | -43.6 |
| Model composition  Non-hydrogen atoms  Protein residues  Ligands | 3294  432  na |
| *B* factors (Å^2^)  Protein  Ligand | 48.4  na |
| R.m.s. deviations  Bond lengths (Å)  Bond angles (°) | 0.011  2.348 |
| Validation  MolProbity score  Clashscore  Poor rotamers (%) | 1.06  0  0 |
| Ramachandran plot  Favored (%)  Allowed (%)  Disallowed (%) | 89.76  10.24  0 |

**Table S2:** **Cryo-EM data statistics and model validation of second-generation**

**C322S 0N3R filaments.**

| **LMB Krios III** | **2nd generation**  **C322S 0N3R**  (EMDB 53530)  (PDB 9R2H) |
| --- | --- |
| **Data acquisition** |  |
| Electron gun | FEG |
| Detector | GATAN K3 |
| Energy filter slit (eV) | 20 |
| Magnification | 165,000 |
| Voltage (kV) | 300 |
| Electron dose (e-/Å^2^) | 40 |
| Defocus range (μM) | 0.5 to 2.5 |
| Pixel size (Å) | 0.826 |
| **Data processing** |  |
| Initial particle images (no.) | 2723519 (autopicked Topaz) |
| Final particle images (no.) | 173842 |
| Helical twist (º) | -0.93 |
| Helical rise (Å) | 4.78 |
| Symmetry imposed | C1 |
| Map resolution FSC 0.143 (Å) | 2.6 |
| **Refinement** |  |
| Initial model used (PDB code) | 6hre |
| Model resolution FSC 0.5 (Å) | 2.5 |
| Map sharpening *B* factor (Å^2^) | -66 |
| Model composition  Non-hydrogen atoms  Protein residues  Ligands | 2649  357  na |
| *B* factors (Å^2^)  Protein  Ligand | 41.4  na |
| R.m.s. deviations  Bond lengths (Å)  Bond angles (°) | 0.011  2.055 |
| Validation  MolProbity score  Clashscore  Poor rotamers (%) | 0.82  0  0 |
| Ramachandran plot  Favored (%)  Allowed (%)  Disallowed (%) | 95.44  4.56  0 |
